# Ischaemic Strokes in Patients with Pulmonary Arteriovenous Malformations and Hereditary Hemorrhagic Telangiectasia: Associations with Iron Deficiency and Platelets

**DOI:** 10.1371/journal.pone.0088812

**Published:** 2014-02-19

**Authors:** Claire L. Shovlin, Basel Chamali, Vatshalan Santhirapala, John A. Livesey, Gillian Angus, Richard Manning, Michael A. Laffan, John Meek, Hannah C. Tighe, James E. Jackson

**Affiliations:** 1 National Heart & Lung Institute (NHLI) Cardiovascular Sciences, Imperial College London, United Kingdom; 2 Respiratory Medicine, Imperial College Healthcare NHS Trust, London, United Kingdom; 3 Imperial College School of Medicine, Imperial College London, United Kingdom; 4 Haematology, Imperial College Healthcare NHS Trust, London, United Kingdom; 5 Centre for Haematology, Investigative Sciences, Imperial College London, United Kingdom; 6 Clinical Chemistry, Imperial College Healthcare NHS Trust, London, United Kingdom; 7 Department of Imaging, Imperial College Healthcare NHS Trust, London, United Kingdom; Odense University Hospital, Denmark

## Abstract

**Background:**

Pulmonary first pass filtration of particles marginally exceeding ∼7 µm (the size of a red blood cell) is used routinely in diagnostics, and allows cellular aggregates forming or entering the circulation in the preceding cardiac cycle to lodge safely in pulmonary capillaries/arterioles. Pulmonary arteriovenous malformations compromise capillary bed filtration, and are commonly associated with ischaemic stroke. Cohorts with CT-scan evident malformations associated with the highest contrast echocardiographic shunt grades are known to be at higher stroke risk. Our goal was to identify within this broad grouping, which patients were at higher risk of stroke.

**Methodology:**

497 consecutive patients with CT-proven pulmonary arteriovenous malformations due to hereditary haemorrhagic telangiectasia were studied. Relationships with radiologically-confirmed clinical ischaemic stroke were examined using logistic regression, receiver operating characteristic analyses, and platelet studies.

**Principal Findings:**

Sixty-one individuals (12.3%) had acute, non-iatrogenic ischaemic clinical strokes at a median age of 52 (IQR 41–63) years. In crude and age-adjusted logistic regression, stroke risk was associated not with venous thromboemboli or conventional neurovascular risk factors, but with low serum iron (adjusted odds ratio 0.96 [95% confidence intervals 0.92, 1.00]), and more weakly with low oxygen saturations reflecting a larger right-to-left shunt (adjusted OR 0.96 [0.92, 1.01]). For the same pulmonary arteriovenous malformations, the stroke risk would approximately double with serum iron 6 µmol/L compared to mid-normal range (7–27 µmol/L). Platelet studies confirmed overlooked data that iron deficiency is associated with exuberant platelet aggregation to serotonin (5HT), correcting following iron treatment. By MANOVA, adjusting for participant and 5HT, iron or ferritin explained 14% of the variance in log-transformed aggregation-rate (p = 0.039/p = 0.021).

**Significance:**

These data suggest that patients with compromised pulmonary capillary filtration due to pulmonary arteriovenous malformations are at increased risk of ischaemic stroke if they are iron deficient, and that mechanisms are likely to include enhanced aggregation of circulating platelets.

## Introduction

Who is most at risk of paradoxical embolic strokes through the right-to-left shunts provided by pulmonary arteriovenous malformations (PAVMs)[1]? This question is important not only for patients, but also to further our understanding of pulmonary capillary filtration.[2] [3]

**Figure 1:**
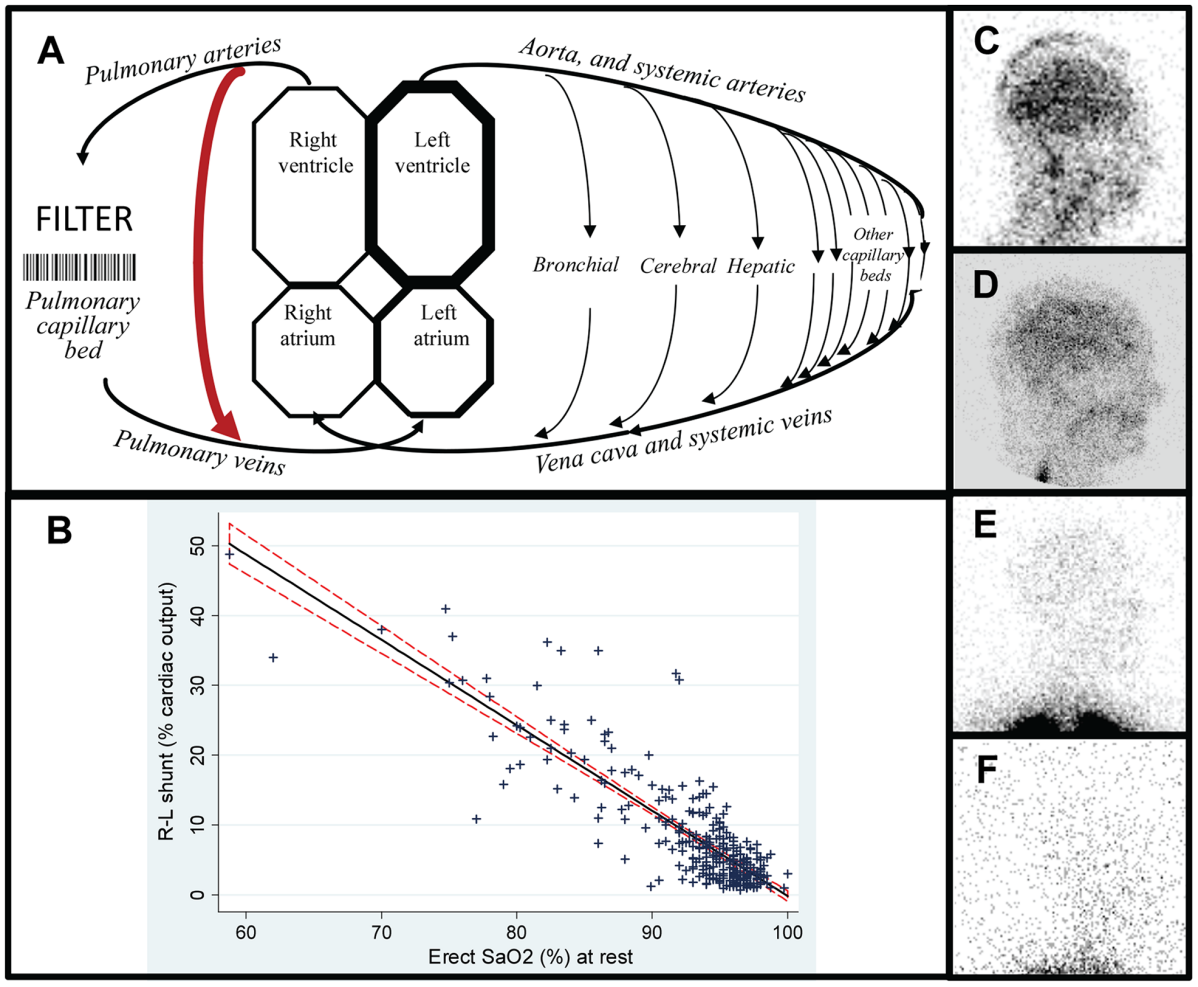
Right-to-left shunt and hypoxaemia evaluations. **A**: Cartoon of the circulations indicating site of the pulmonary capillary filter, the dual pulmonary and bronchial/systemic arterial supply to lung tissue, and a pulmonary arteriovenous malformations (PAVM, red arrow). **B**: Relationship between quantified right-to-left shunt (measured using with ^99m^Tc-labelled albumin macroaggregates (10–80 µm) or microspheres (7–25 µm)), with same-day oxygen saturation (SaO_2_), representing 309 paired values in 198 individuals since 1999. The linear regression coefficient of −1.22 (95% CI −1.31, −1.14; p<0.0001) indicates a strong relationship that explains 73% of the total variance in erect SaO_2_ (adjusted r^2^ 0.73). The shunt explained a smaller proportion of the total variance in supine SaO_2_ (adjusted r^2^ 0.54, data not shown). **C–F**: Representative right lateral brain images following injection of ^99m^Tc-labelled albumin macroaggregates for shunt diagnosis and quantification: **C**) R-L shunt 48.8% of the cardiac output, associated with a resting SaO_2_ of 59%. **D**) R-L shunt 25%; SaO_2_ 83%. **E**) R-L shunt 7.7%; SaO_2_ 93.7%. Note the intense activity in the lung apices as expected. **F**) R-L shunt 3.3%; SaO_2_ 96%. Note that the gain has been turned up but no cerebral activity is visible. This is the same individual as in **D**), with the images taken 6 months before (**D**) and 3 months after (**F**) embolisation which obliterated the causative PAVMs.

The pulmonary capillary bed normally provides a first-pass filtration system for particulate matter forming or entering the circulation in the preceding cardiac cycle: lung viability is preserved due to dual arterial supply from systemic arteries, particularly the bronchial circulation (Figure 1A). [1] [2] [3] Pulmonary capillary filtration is used routinely in clinical diagnostics, using intravenous injection of technetium-labelled albumin macroaggregates marginally larger than 7 µm erythrocytes for nuclear medicine perfusion studies, [4] [5] or bubble contrast in echocardiography.[6]

PAVMs are anatomical right-to-left shunts between pulmonary arteries and pulmonary veins, [1] (Figure 1), and by thoracic CT scanning, are estimated to affect ∼1/2,600 individuals. [7] Right-to-left shunts, including intrapulmonary shunts below the resolution of CT-scan detection, can be quantified by the circulatory survival of intravenously injected particles: particles transiting the shunts bypass the pulmonary capillaries, and reach the left ventricle and systemic arterial tree. Technetium-labelled albumin macroaggregates as used for conventional perfusion studies permit precise quantification of right-to-left shunt size as a proportion of the cardiac output. [4] [8] [9] [10] [11] Contrast echocardiography can provide broad grades of shunt severity, ranging from Grade 1 (found in at least 7–8% of the general population [12] [13]), to Grade 3 which is more frequently associated with visible PAVMs on CT scan. [13[14] [15] [16] [17] [18] Additionally, because PAVMs allow deoxygenated pulmonary arterial blood to bypass the pulmonary capillary bed, arterial PO_2_ and haemoglobin saturation (SaO_2_) are inversely related to the size of the right-to-left shunt fraction.[1] [4] [9] [10]

Substantial proportions of PAVM patients experience ischaemic strokes. [1] [13] [18] [19] [20] [21] Paradoxical embolic events are more common in patients with Grade 3 shunts, the shunt severity usually present when PAVMs are visible on CT scan. [13[14] [15] [16] [17] [18] Once PAVMs are sufficiently large for CT detection, or Grade 3 shunts, there is no clear evidence that stroke risk is substantially influenced by further increase in shunt size,[18] [20] or by conventional stroke risk factors.[20] Understanding which PAVM patients have their strokes at particular times is important because while PAVM treatment by embolisation reduces stroke risk, [20] PAVMs are often technically too small for embolisation, and many treated patients are left with residual right-to-left shunts.[1] [20]

The majority of PAVMs occur as part of hereditary hemorrhagic telangiectasia (HHT) [1] [4] [11] [13] [18] [19] [20] [21] This vascular condition [22] [23] [24] is usually caused by mutations in *endoglin (ENG* **,** HHT type 1), [25] *ACVRL1/ALK1* (HHT type 2), [26] or *Smad4* (HHT-juvenile polyposis).[27] PAVMs affect approximately 50% of HHT patients [21] and are particularly common in HHT1, with 85% of *ENG* mutation carriers demonstrating right-to-left shunts on contrast echocardiography. [28] HHT also commonly results in hepatic, [29] cerebral [30] and other visceral AVMs; mucocutaneous telangiectasia that lead to epistaxis [31] [32] and gastrointestinal bleeding [33]; and iron deficiency which has been recently confirmed to result from under-replacement of haemorrhagic iron losses.[34] At the outset of this study, there was no indication that any aspect of HHT pathology other than PAVM-associated factors would be related to stroke risk. The general presumption was that ischaemic strokes developed as a result of paradoxical emboli of venous thromboemboli (VTE) either from the systemic venous circulation, [35] or arising in the PAVM sac, [36] and that stroke risk would increase with the severity of PAVMs.[19]

The study objectives, to identify new risk factors for ischaemic stroke in PAVM patients, were achieved. Here, focusing on PAVM patients with underlying HHT, we provide new insights into which PAVM patients more commonly have ischaemic strokes.

## Methods

All studies were conducted according to the principles expressed in the Declaration of Helsinki. Ethical approved was from the Hammersmith, Queen Charlotte's, Chelsea, and Acton Hospital Research Ethics Committee. For LREC 2000/5764: “ Case Notes Review: Hammersmith Hospital patients with pulmonary arteriovenous malformations and hereditary haemorrhagic telangiectasia (HHT),” the ethics committee approved the review of the case notes for research purposes without seeking individual consents. For LREC 2000/6308 “Studies of blood cells derived from HHT Patients”, individuals provided written consent for the blood tests which were not part of standard clinical practice.

The study objective was to identify further risk factors for ischaemic stroke in PAVM patients. Our previous studies in a PAVM population in which 205/219 (93.6%) had HHT, demonstrated an ischaemic stroke rate of 13.7%. [20] A population of 450 would therefore have 90% power to detect a difference of 0.45 standard deviations for tested variables at the 5% significance level (two-tailed). As it was not clear whether such stroke rates and hence power calculations would apply to PAVM cohorts with a greater number of sporadic cases, for the current study, analyses were restricted to PAVM patients with HHT.

### Study Cohort

The cohort represents 497 consecutive adult PAVM patients with a clinical diagnosis of HHT,[37] and radiologically-confirmed PAVMs, reviewed May 1999-February 2013 in a pan-UK tertiary service (www.imperial.ac.uk/nhli/hht_pavm_patient).

Assessments were performed in keeping with clinical service practice. As detailed below, based on existing protocols and a systematic literature review [38], from the outset of the study in 1999, individuals presenting with definite or possible HHT [37] and/or PAVMs underwent detailed same-day clinical assessments including imaging (chest x-ray and thoracic CT scans if not performed previously); pulmonary function testing including measurement of oxygen saturation by pulse oximetry (SaO_2_); blood tests; and shunt studies, before subsequent embolisation of PAVMs where appropriate. Over the 14 years of the study, routine clinic protocols were updated according to new published evidence, and results of additional research study protocols.

### Standard Clinical Assessments

#### Imaging

Chest x-rays were performed for all patients on presentation and in follow up. At the outset of the study in 1999, based on a literature review of published PAVM cases [38] and established service practices,[10] thoracic CT scans were only performed if there were relevant symptoms (respiratory or neurological such as transient ischaemic attacks, strokes or brain abscess); or if there was evidence of hypoxaemia or a rightto-left shunt by screening methods (see below). By 2002, it was apparent that very small PAVMs with feeding artery diameters <3 mm could be associated with ischaemic stroke and/or brain abscess,[20] and CT scans were introduced as routine screening for all patients, if not performed previously, initially using adapted Remy protocols.[39] The radiation burden has been substantially decreased, and the resolution improved, by the use of newer multislice, multidetector CT, which limit x-ray exposure to a single short breath-hold acquisition, and which allow multiplanar and three dimensional reconstructions of the data.[40]

Although we were aware that other PAVM groups internationally were introducing contrast echocardiography as a screening tool, [41] and research studies were performed, [42] contrast echocardiography was not introduced routinely into the service for PAVM screening. As discussed at the time, [43] [44] this was because of the high frequency of apparent false negatives where PAVMs were not identified by subsequent angiography, considered to be the gold standard for detection of PAVMs (10/25 cases in [41]; 20% in [45] and 6/11 in [46]); the frequency with which the studies were positive requiring subsequent CT evaluations [1] [44]; service logistics; patient preference for the speed and cannula free CT scan; research study variability in grey scale and spectral Doppler of the carotid artery post Echovist injection on repeated studies; [42] [47] limited correlations with validated methods of right-to-left shunt quantification; [42] [47] and provocation of angina with electrocardiographic ST segment depression in a patient with a large right-to-left shunt. These research studies were never formally published due to the untimely death of the late Professor Martin Blomley.[48]

#### Right-to-left (R-L) shunt measurements

These were routinely made at the outset of the study using established departmental protocols. [4] [8] [9] [10] [49] [50] For these nuclear medicine quantifications, 71–150 MBq (median 100 [IQR 93,106]) MBq of ^99m^technetium-labelled albumin macroaggregates were injected into the antecubital vein. The shunt was formally quantified by the proportion of radioactive tracer detected over the right kidney (posterior view), adjusted for dose injected, and assuming the right kidney receives 10% of cardiac output (kidney dose method). [4] [11] By 2002, it was apparent that very small PAVMs with right-to-left shunts below the usual diagnostic cut off [10] could be associated with strokes and brain abscess: this was formally confirmed as a lack of association with paradoxical embolic endpoints. [20] Routine right-to-left shunt measurements were phased out after 2006 in view of these data; the cerebral images (Figure 1C–F); patient reports of headaches provoked by the scans; and further validations of oxygen quantification methodologies.

#### Other clinical assessments

General clinical variables defined at the time of initial clinic assessment included a full symptomatic review [51]; full past medical, [20] [52] pregnancy, [53] and family [53] [54] histories; histories of current or ex-smoking; full drug history with a particular focus on treatments used for HHT haemorrhage (in this series, female hormones, tranexamic acid and iron/blood replacement (oral and intravenous iron; blood transfusions)); hypertension on treatment or blood pressure >140/90; atrial fibrillation on treatment or ECG; and known diabetes mellitus, hypercholesterolaemia or cardiac disease. Headaches were defined as migrainous for patients on migraine treatment, or describing recurrent headaches with aura and/or teichopsia.[55]

Strokes were defined as clinically evident, focal cerebral deficits of rapid onset, at least 24 hs in duration. This population have heightened susceptibility to other strokes and stroke-like pathologies [1] [12] [13] [14]. We have previously addressed haemorrhagic strokes, [54] brain abscesses,[20] [56] and migraines, [55] in the population. For the current study which focused specifically on ischaemic strokes, an independent report of a causative infarct on clinical post stroke cross-sectional imaging was required to assign ischaemic aetiology.

Polycythaemia, and anaemia due to iron deficiency, are common problems in PAVM and HHT patients. [14] [15] [16] [51] Throughout the study, blood tests included full blood counts with haematinics, routine biochemistry, coagulation screens, and iron assessments (serum iron and transferrin saturation index (T*f*SI)). To optimise clinical management, blood tests were taken at standardised times to minimise variability due to diurnal variation in iron levels, which at the study outset was anticipated to lead to daytime falls in iron [57] Following the identification of venous thromboemboli risks and elevated FVIII in a research study protocol [52], Factor VIII:Ag was included in routine blood tests from 2002, but to avoid confounding variables, not within 6 months of a known confounding state such as VTE, infection, therapeutic embolisation, surgery or pregnancy.[52] [57]

Our interim 2006 analyses demonstrated multiple associations between clinically relevant endpoints and iron treatment and/or iron deficiency (Shovlin, Jackson, and Kulinskaya, 2006 unpublished). Recognising that identifying incorrect associations could lead to potentially detrimental clinical care, the iron deficiency/treatment associations were not published in the relevant manuscripts. [20] [52] [58] [59] Instead, extensive literature searches to identify potential mechanistic routes were undertaken, and relevant clinical research study protocols performed to define why iron deficiency was present; [34] examine relationships with hypoxaemia compensations; [51] and explore potential mechanisms for associations with thrombotic and other clinical endpoints ([60] CIBA Foundation searches; current manuscript; Mollet et al manuscript in preparation). For the clinical service, ferritin, which had not been measured initially due to concerns about interpretation in populations at high risk of hepatic AVMs,[61] [62] was measured routinely from 2006.

#### Embolisation

262 (62.1%) patients underwent embolisation treatment of PAVMs, [40] [58] [63] with embolisation not indicated in the remainder primarily due to PAVMs with feeding arteries too small for treatment. Other reasons for no embolisation include previous maximal treatment elsewhere, contraindications (four with very severe pulmonary hypertension,[58] with one on a liver transplant waiting list), or patient preference. Pulmonary artery pressures (PAP) were measured in 262 patients undergoing embolisation, recorded by a centrally-placed catheter prior to contrast injection.[40] [58] [63]

### Study Specific Methods

#### Oxygen saturation and validations

Pulse oximetry had been introduced into the PAVM clinical service more than a decade earlier [10] because of patient preference compared to the repeated arterial punctures required to obtain arterial blood gases. Pulse oximetry at 1 minute intervals for 10 minutes standing, with the mean of the recordings at minutes 7, 8, 9 and 10, had been validated as a robust measure of PAVM severity, better reflecting right-to-left shunt size than SaO_2_ in other postures, or on exercise.[10]

For the current study, Table 1 demonstrates that the replicate four values following 7, 8, 9 and 10 minutes standing were highly reproducible. To further validate the mean value for use in the current manuscript, the mean SaO_2_ was compared in the subgroup of patients with same-day right-to-left shunt measurements. Same-day shunt measurements were available in 198 cases and ranged from 0.7–48.8% of cardiac output (median 5.3%). The right-to-left shunt explained 73% of the total variance in mean SaO_2_ (Figure 1B).

**Table 1:** SaO_2_ measurement reproducibility.

	*Overall*	*Quintile 1*	*Quintile 2*	*Quintile 3*	*Quintile 4*	*Quintile 5*
Number of datasets	522	104	104	104	104	103
SaO_2_ erect (%): mean (SD)	93.9 (4.1)	86.9 (3.7)	93.2 (1.0)	95.4 (0.72)	96.5 (0.6)	97.3 (0.68)
SD of 4 replicates (%): mean (SD)	0.40 (0.44)	0.60 (0.74)	0.37 (0.29)	0.34 (0.30)	0.30 (0.31)	0.38 (0.30)

SaO_2_ values measured by pulse oximetry following 7, 8, 9 and10 minutes standing. The variability within these measurements has not been presented previously. 522 consecutive datasets for the 165 PAVM patients first presenting between 2006 and 2010 were analysed and represent their datasets at presentation and in follow up. To illustrate reproducibility across all severities of hypoxaemia, datasets were divided into quintiles based on SaO_2_, each with over 100 datasets. SD, standard deviation.

#### Blood tests and validations

Iron deficiency is a common problem in HHT patients [14] [15] [16] A low serum ferritin defines iron deficiency, but as discussed previously by ourselves [60] and others, [61] [62] iron deficiency may be present in the setting of a normal or high ferritin due to concurrent pathologies: Inflammation, liver pathology, kidney disease, malignancy, rheumatoid disease, hyperthyroidism, heavy alcohol intake, and hepatic AVMs are all associated with high ferritin concentrations despite severe iron deficiency. [60] [61] [62] Additionally, iron deficiency may be difficult to assess in the PAVM/HHT patients, because secondary polycythaemia in hypoxaemic patients masks anticipated anaemia. [35] [51] [60] To evaluate if low serum iron levels were capturing similar iron deficient cohorts to low serum ferritin, haematinic variables were examined in patients with ferritin<15 µg/L, ferritin<20 µg/L, or serum iron <11 µmol/L irrespective of ferritin. As shown in Table 2, the iron-deficient haematinic variables were indistinguishable between these three iron-deficiency definitions, and all differed substantially from the group without evidence of iron deficiency. We concluded that it was appropriate to use low serum iron, measured during the standardised afternoon timepoints, as a marker of a clinically relevant iron deficient state.

**Table 2:** Comparison of iron deficiency classifications.

	Median				Lower quartile (Q1)				Upper quartile (Q1)			
	No ID	ferr<15	ferr <20	iron <11	No ID	ferr<15	ferr <20	iron <11	No ID	ferr<15	ferr <20	iron <11
Age (yr)	48	47.5	47.5	52	35	37.5	39	43	62	52.5	54	61
SaO_2_ (%)	95	95	94.6	94.9	92	90.5	89.3	92	96.5	96	95.5	95.8
BMI (kg/m^2^)	25.8	25.1	25.3	26.3	22.9	21.9	21.9	21.9	28.2	28.3	29.8	30.2
Haemoglobin (g/dl)	15.1	**12.8**	**12.9**	**13**	13.9	**11.6**	**12.3**	**11.9**	16	**13.7**	**13.8**	**14.2**
Haematocrit	0.44	**0.4**	**0.4**	**0.4**	0.41	**0.38**	**0.39**	**0.38**	0.47	**0.42**	**0.43**	**0.44**
MCV (fl)	89.6	**84.8**	**85**	**85**	86.8	**73.4**	**73.9**	**76.4**	92.8	**86.9**	**87.4**	**87.4**
MCH (pg)	30.7	**26.6**	**27.2**	**27.6**	29.4	**22.6**	**22.8**	**23.9**	31.8	**29**	**29.1**	**29.2**
MCHC (g/dL)	34	**32.1**	**32.2**	**32**	33.3	**30.7**	**30.6**	**31**	34.7	**33.1**	**33.4**	**33.2**
Serum iron (µmol/L)	18	**7**	**8.5**	**7**	16	**4**	**4**	**4**	24	**13**	**13**	**10**
Serum T*f*SI (%)	30.5	**10.5**	**15**	**11**	27	**6**	**6**	**6**	44	**17.5**	**21**	**15**
Serum ferritin µg/L	51	7	9.5	19	31.5	**5.5**	**6**	**7**	93	10.5	14	40
Oxygen content (mls/dl)	18.8	**16**	**16**	**16.3**	17.4	**14.9**	**14.8**	**14.8**	20.1	**17.4**	**17.6**	**18.1**

For patients presenting between 2006 and 2010, iron deficiency was assigned as absent (No ID) if ferritin, iron and T*f*SI were all clearly in the normal range (ferritin>20 µg/L, serum iron>11 µmol/L and T*f*SI>20%, N = 93). The groupings to examine iron deficiency were ferritin <15 µg/L (ferr<15, N = 20); ferritin <20 µg/L (ferr<20, N = 26); and serum iron <11 µmol/L (iron <11, N = 50). BMI, body mass index. MCV, mean corpuscular volume. MCH, mean corpuscular haemoglobin. MCHC, mean corpuscular haemoglobin concentration. T*f*SI, transferrin saturation index.

#### Platelet aggregation

Following identification of a potentially highly relevant manuscript describing modified platelet aggregation responses to serotonin (5 hydroxytryptophan, 5HT) in iron deficient patients[64] platelet aggregation responses were examined in a subgroup of HHT patients. The maximal % aggregation, and slope representing rate of platelet aggregation were measured with light transmission (Born) aggregometry (Helena Bioscience APACT4) in platelet rich plasma (PRP) within 2 hours of blood sampling: 10 mls of blood from consenting individuals was collected into citrate tubes, and centrifuged at 900 rpms for 10 mins to generate platelet rich plasma. To generate platelet poor plasma, the remaining blood was centrifuged at 3,000 rpms for a further 10 mins. The turbid platelet rich plasma and clear platelet poor plasma were used for APACT4 turbidity reference settings representing 0 and 100% aggregation respectively. For experimental assays, fresh solutions of adenosine diphosphate (ADP, Helena Biosciences), and serotonin (5HT, Sigma) were prepared, and serial dilutions used to generate working concentrations. For ADP these were 50 µM, 20 µM, 10 µM, and 5 µM; for 5HT 20,000 µM, 2,000 µM, 400 µM, 200 µM and 20 µM. For aggregation assays, 25 µl of agonist was added to 225 µl of platelet rich plasma, and aggregation over 300 s measured by the loss in plasma turbidity as platelets aggregated and sedimented. All measured read outs were entered into an Excel database and blood test values added, before transferring to STATA for statistical analyses which used absolute iron/ferritin values (for graphical illustration, patients were divided into groups).

Very careful patient selection was used to permit utilisation of serum ferritin, because in Woods et al, [64] platelet aggregation reflected iron status over preceding weeks and not days. Multiple disease states are associated with high ferritin concentrations despite severe iron deficiency both in the general population, [61] [62] and in HHT patients [60] who have normal iron handling and appropriate hepcidin levels. [34] Potential participants were therefore selected if (based on current nosebleeds[65] and prior history/investigations), they were anticipated to be consistently iron deficient or non iron deficient, not taking anti-platelet agents, [66] not transfused or receiving intravenous iron, [60] and without known concurrent inflammatory or hepatic disease. [60] [61] [62] Due to logistics of same-day blood sampling and processing, platelet assays were performed before the routine ferritin and iron results were known.

#### Statistical analyses

Statistical analyses were performed using STATA IC version 12 (Statacorp, Texas), with adjustments for multiple testing performed using the false discovery rate (FDR).[67] Datasets utilised presentation values (non stroke patients), or timepoint closest to the stroke (stroke patients). Age/gender and iron-adjusted odds ratios were calculated by adding each variable to separate models which were simultaneously examining the associations of first ischaemic stroke with age and gender, or serum iron: *p* values were calculated by post-estimation Wald tests. Final models were confirmed using receiver operating characteristic (ROC) analyses. Variance contributions in multivariate analysis of variance (MANOVA) were calculated using Wilks' lambda.

## Results

### Population demographics

Full demographics of the series are presented in Table 3. The median age of the 497 consecutive and unselected PAVM/HHT patients was 47 (IQR 35, 60)ys. 302(60.8%) were female. 231(46.4%) had recognisable neurovascular risk factors, including 185(37.2%) current/former smokers, 75(15.1%) with documented or treated hypertension, and smaller numbers with hypercholesterolaemia, diabetes, or arrhythmias. SaO_2_ ranged from 59–100% (median 94.75%, IQR 91,96%). Due to HHT bleeding, predominantly from the nose, [34] 137(28%) had used iron tablets, 17(3.5%) had used intravenous iron, and 42(8.6%) had been transfused.

**Table 3:** Population Demographics.

	*No stroke*			*Ischaemic stroke*			*Total*			*Mann Whitney*
	*N*	*median*	*mean*	*N*	*median*	*mean*	*N*	*median*	*mean*	*p value*
Ischaemic stroke	436	0	0	61	1	1	497	0	0.12	—
Age (yr)	436	46	46.4	61	51	51.5	497	47	47.0	**0.025**
Gender (% female)	436	1	0.60	61	1	0.69	497	1	0.61	0.17
Smoking	436	0	0.37	61	0	0.38	497	0	0.37	0.94
Hypertension	434	0	0.14	61	0	0.21	495	0	0.15	0.15
Venous thromboemboli	436	0	0.053	61	0	0.049	497	0	0.052	0.91
Diabetes mellitus	436	0	0.011	61	0	0.033	497	0	0.014	0.19
Atrial fibrillation or arrythmia	436	0	0.014	61	0	0.016	497	0	0.014	0.87
Hypercholesterolemia	435	0	0.016	61	0	0.016	496	0	0.016	0.97
Ischaemic heart disease	436	0	0.0046	61	0	0.049	497	0	0.01	**0.0011**
Myocardial or valvular disease	436	0	0.0092	61	0	0	497	0	0.008	0.45
High output cardiac failure	436	0	0.0046	61	0	0	497	0	0.004	0.6
Brain abscess	436	0	0.096	61	0	0.098	497	0	0.10	0.96
Migraines	435	0	0.35	61	0	0.33	496	0	0.35	0.74
Ever transfused (%)	430	0	0.081	57	0	0.12	487	0	0.086	0.3
On iron treatment (oral)	431	0	0.27	58	0	0.36	489	0	0.28	0.14
Ever used intravenous iron	425	0	0.033	57	0	0.053	482	0	0.035	0.45
Ever used tranexamic acid	425	0	0.028	57	0	0.035	482	0	0.029	0.77
Ever used hormones	423	0	0.10	57	0	0.16	480	0	0.11	0.22
Oxygen saturation (SaO_2_, %)	414	95	92.9	57	94	91.8	471	94.8	92.7	0.28
Hemoglobin (g/dl)	404	14.2	15.4	60	13.8	17.5	464	14.1	15.7	0.25
Platelets (x10^9^/dl)	405	267	275.5	60	272.5	284.8	465	268	276.7	0.57
Serum iron (µmol/L)	364	12	13.7	55	9	10.7	419	12	13.3	**0.025**
Serum transferrin saturation index, T*f*SI(%)	364	20	21.7	56	15	18.4	420	19	21.2	0.094
Ferritin (µg/L)	214	29.5	49.5	28	34.5	49.8	242	30	49.6	0.55
C-reactive protein, CRP (iu/ml)	275	2	3.49	35	2	7.25	310	2	3.91	0.59
Prothrombin time, PT (s)	385	10.7	10.9	54	10.5	11.2	439	10.7	11.0	0.08
Activated partial thromboplastin time, APTT (s)	381	26	26.1	54	25.65	25.8	435	26	26.1	0.27
Thrombin time, TT (s)	369	14.6	14.6	52	15	15.1	421	15	14.7	0.07
Fibrinogen (g/L)	381	3.03	3.11	58	3.34	3.48	439	3.07	3.16	**0.013**
Factor VIII:Ag, FVIII (iu/ml)	240	1.50	1.63	40	1.73	1.87	280	1.52	1.66	0.07
Von Willebrand Factor, VWF (iu/ml)	177	1.01	1.25	25	1.1	3.00	202	1.025	1.47	0.36
Pulmonary artery pressure (systolic), PAP, mmHg	217	23	24.1	45	23	24.1	262	23	24.1	0.69
Pulmonary artery pressure (diastolic), PAP, mmHg	217	8	8.2	45	7	7.82	262	7	8.16	0.63
Pulmonary artery pressure (mean), PAP, mmHg	214	14	14.5	45	13	14.0	259	14	14.4	0.51
Series Indicator	436	2	1.8	61	1	1.63	497	2	1.75	0.13

Demographics of individual and combined series, with p values calculated by Mann Whitney. N, number of recordings of variable in series.

### Paradoxical embolic events

No neurological deficits or myocardial ischaemic symptoms were reported by the patients following perfusion study quantification of the right-to-left shunt. However, larger shunts were associated with substantial cerebral microvascular impaction, and this reduced after PAVM embolisation (Figure 1C–F).

Sixty-one individuals (12.3%) experienced at least one clinical stroke confirmed as ischaemic in aetiology at median age 52 ys (IQR 41–63 ys). By Oxford/Bamford classification, 43/61 (70.5%) were partial anterior circulation syndromes, 17/61 (27.9%) were partial posterior circulation syndromes, and one patient had a lacunar circulation syndrome (Table 4). There was no difference in ages between the stroke distribution types (Table 4).

**Table 4:** Details of clinical ischaemic strokes.

		*N*	*Gender (% female)*	*Age at stroke median (range)*
Total anterior circulation	TACS	0	—	----
Partial anterior circulation	PACS	43	65·1	51 (24–77)
Partial posterior circulation	POCS	17	53·1	52 (25–89)
Lacunar circulation*	LACS	1	100	58
Total	ALL	61	51·5	51 (24–89)

*Note that silent lacunar infarcts, or silent infarcts at other sites, were excluded by the study methodology. Data exclude four iatrogenic strokes of known aetiology (one following thrombus injection through giving set; one at time of cerebral angiography; one at time of pulmonary angiography; and one progressive following stereotactic radiotherapy), but otherwise include all first clinical strokes of ischaemic aetiology.

### Conventional stroke risk factors

Almost half of the ischaemic strokes (29/61, 47.5%) occurred in lifelong non smokers without any documented conventional stroke risk factors (hypertension; hypercholesterolemia, diabetes, or arrhythmias). Ischaemic strokes were no more frequent in patients with one or more conventional risk factors (32/231) than those without any risk factors (29/266, χ^2^ p = 0·42). In age/gender-adjusted logistic regression, none of the conventional neurovascular risk factors were associated with ischaemic stroke risk (Table 5).

**Table 5:** Adjusted odds ratios for ischaemic stroke risk.

	Age and gender-adjusted			Serum iron-adjusted		
	*N*	*Odds Ratio*	*p value*	*N*	*Odds Ratio*	*p value*
Age	—	—	—	419	1·02 (1·00, 1·03)	0·13
Gender	—	—	—	419	1·40 (0·75, 2·58)	0·29
Smoking	497	1·08 (0·62, 1·91)	0·78	419	0·94 (0·52, 1·70)	0·84
Hypertension	495	1·32 (0·65, 2·67)	0·44	417	1·27(0·60, 2·68)	0·54
Diabetes	497	3·34 (0·61, 18·2)	0·16	419	3·10 (0·54, 17·6)	0·2
AF and other arrythmias	497	1·05 (0·12, 9·21)	0·96	419	1·07 (0·12, 9·56)	0·95
Hypercholesterolemia	496	0·78 (0·09, 6·65)	0·82	418	1·24 (0·14, 10·6)	0·85
Myocardial infarction (MI)	497	8·86 (1·41, 55·5)	0·02	419	22·6 (2·26, 226·7)	0·008
Brain abscess	497	0·98 (0·39, 2·46)	0·97	419	0·88 (0·33, 2·35)	0·02
Migraines	496	0·87 (0.49, 1·56)	0·43	418	0·89 (0·49, 1·62)	0·71
Venous thromboemboli (VTE)	497	0·77 (0·22, 2·67)	0·68	419	0·53 (0·11, 2·34)	0·4
Ever transfused	487	1·28 (0·53, 3·12)	0·58	413	1·46 (0·60, 3·58)	0·41
On iron treatment (oral)	489	1·28 (0·70, 2·34)	0·41	413	1·14 (0·60, 2·15)	0·68
Ever used intravenous iron	482	1·41 (0·39, 5·17)	0·6	407	1·31 (0·35, 4·85)	0·69
Ever used tranexamic acid	482	0·99 (0·21, 4·6)	0·99	407	1·22 (0·25, 5·98)	0·8
Ever used female hormones	480	1·41 (0·64, 3·14)	0·4	405	1·47 (0·64, 3·40)	0·37
Oxygen saturation erect (SaO_2_)†	471	0·98 (0·93, 1·02)	0·28	398	0·96 (0·92, 1·01)	0·085
Haemoglobin (g/dl)	464	1·00 (0·99, 1·02)	0·38	410	0·98 (0·91, 1·05)	0·62
Platelets	465	1·00 (1·00, 1·00)	0·67	415	1·00 (1.00, 1.00)	0·48
Serum iron †	419	0·96 (0·92, 1·00)	0·036	—	—	—
Transferrin saturation index (T*f*SI)	420	0·99 (0·97, 1·01)	0·17	418	1·00 (0·95, 1·06)	0·93
Ferritin	242	1·00 (0·99, 1·01)	0·91	236	1·00 (0·99, 1·01)	0·74
C-reactive protein	310	1·03 (1·00, 1·07)	0·06	292	1·03 (0·99, 1·06)	0·1
Prothrombin time	439	1·08 (0·96, 1·21)	0·2	394	1·02 (0·87, 1·20)	0·83
Activated partial thromboplastin time	435	0·97 (0·87, 1·07)	0·54	391	1·00 (0·90, 1·11)	0·97
Thrombin time	421	1·14 (0·97, 1·38)	0·11	379	1·13 (0·96, 1·34)	0·14
Fibrinogen	439	1·24 (1·00, 1·53)	0·048	393	1·16 (0·91, 1·47)	0·25
Factor VIII	280	1·45 (0·95, 2·30)	0·089	263	1·24 (0·78, 1·96)	0·36
von Willebrand Factor	202	1·09 (0·97, 1·23)	0·14	188	0·99 (0·70, 1·40)	0·95
Pulmonary artery pressure (systolic)	262	0·99 (0·94, 1·04)	0·71	221	0·97 (0·92, 1·03)	0·39
Pulmonary artery pressure (diastolic)	262	0·96 (0·88, 1·06)	0·45	221	0·96 (0·87, 1·06)	0·44
Pulmonary artery pressure (mean)	259	0·96 (0·89, 1·04)	0·3	218	0·94 (0·86, 1·03)	0·17
Series indicator	497	0·79 (0·54, 1·18)	0·23	419	0·73 (0·48, 1·10)	0·13

Full list of age/gender, and serum iron-adjusted odds ratios for ischaemic stroke risk for the specified variable, where an inverse association with stroke risk is indicated by an odds ratio <1. N: number of datasets available. CI, confidence intervals, AF, atrial fibrillation. Pseudo r^2^ indicates the proportion of stroke variance explained by age, gender, and the specified variable. P values were calculated by the non parametric Wald test which does not assume independence of variables. † Quadratic regression plots for stroke risk versus serum iron or SaO_2_ presented in Figure S1. Note *p* = 0.047 significant at FDR = 0.05 level.

Ischaemic strokes were more common in patients who had experienced at least one myocardial infarction (Table 5). This association persisted after adjustment for all other measured variables, and no other measured variable could replace myocardial infarction in the model, in keeping with a common paradoxical embolic aetiology.[68] However, only five patients had myocardial infarctions, three of whom had also experienced an ischaemic stroke.

Surprisingly, in crude and age/gender adjusted analyses, there was no association between ischaemic stroke and VTE (Table 5).

### Association between ischaemic stroke and iron deficiency

**Figure 2:**
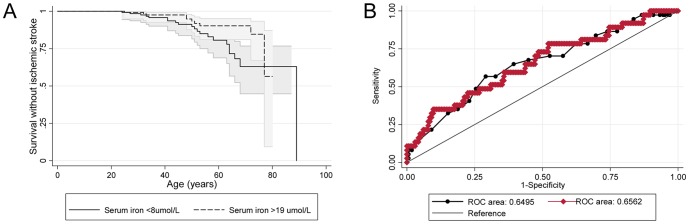
Stroke incidence. **A**) Cumulative survival until first stroke: The solid line is modelled from the 129 patients with serum iron <8 µmol/L; dotted line from the 161 patients with serum iron >19 µmol/L. Shaded areas indicate 95% confidence intervals. **B**) Comparison of the stroke risk ROC models from myocardial infarction and serum iron (base model, black line/symbols), and strongest model generated from captured physiological variables, excluding the outcome measure of myocardial infarction (red line/symbols). The two models provide equivalent areas under the curve of 0.65 and 0·66 (p = 0.88). In the physiological ROC model, stroke risk was higher not only with lower serum iron (OR 0.95 [95% CI 0.90, 1.01]), but also with lower PAP(mean) (OR 0.94 [0.86, 1.03]); higher fibrinogen (OR 1.50 [0.95, 2.33]), lower SaO_2_ (OR 0.98 [95% CI 0.93, 1·03]), and in women (OR 1.57 [0.71, 3.47]).

Ischaemic strokes were more common in patients with low serum iron (Table 5). This association was maintained when adjusted for all other variables. Quadratic regression suggested a near linear inverse relationship between ischaemic stroke risk and serum iron (Figure S1a). The age/gender adjusted odds ratio of 0.96 [95%CI 0.92, 1.00], per µmol/L increase in serum iron, Table 5) implies that a serum iron of 6 µmol/L would be associated with approximately double the risk of stroke compared to a serum iron level in mid-normal range. Iron-stratified Kaplan Meier survival curves are illustrated in Figure 2A.

The study was not powered to evaluate the differences between the groups with and without conventional risk factors, but subgroups analysis suggested that stroke associations with low iron were not confined to patients without known cardiovascular risk factors: The age/gender adjusted odds ratios odds ratios were 0.95 (95%CI 0.90, 1.01, p = 0.075) for the group with known ischaemic stroke risk factors, and 0.97 (95% CI 0.93, 1.03, p = 0.32) for the group without.

### Other associations with stroke risk

It is usually considered that larger right-to-left shunts are associated with an increased risk of neurological complications. Risk are definitely increased in patients with Grade 3 shunts by transthoracic contrast echocardiography (TTCE), compared to shunts of lesser severity.[18] The majority of patients in this study would be expected to have a grade 3 shunt, because they had PAVMs which were visible on CT. [13] [14] [15] [16] [17] [18] Surprisingly, crude analyses demonstrated no association between lower SaO_2_ reflecting larger shunts, and stroke risk (Table 3). In iron-adjusted analyses, there was a marginal association between low SaO_2_ and stroke risk (Table 5): the iron-adjusted odds ratio (0.96 [95%CI 0.92, 1.000]) implied that the risk of stroke would increase ∼1.4 fold with SaO_2_ 90% and ∼2.3 fold with SaO_2_ 80%, for the same degree of iron deficiency.

ROC analyses suggested that in the subgroup of patients with pulmonary artery pressure (PAP) measurements, stroke risk was greater in the setting of lower PAP(mean), supporting our earlier PAVM stroke studies which identified enhanced stroke risk with low PAP. [20] In these ROC analyses, stroke risk was also greater with lower serum iron, lower SaO_2_, higher fibrinogen (the predominant plasma protein for platelet adhesion[69]), and in women (Figure 2B). These associations were evident once adjusted for the presence of all other variables in the ROC model, and therefore making independent contributions to stroke risk in the model.

We concluded that risk factors were pointing away from conventional neurovascular aetiologies, paradoxical embolism of venous thrombi, or shunt severity, towards a process influenced by iron deficiency and paradoxical emboli of platelets.

### Platelet aggregation

Given the established efficacy of anti-platelet therapy in prevention of ischaemic stroke, [70] we hypothesised that iron deficiency may enhance platelet activity. No relevant articles were identified through PubMed. From the CIBA Foundation Symposium back-catalogue, we identified an early report[64] which we believe may have been hitherto underappreciated. Supporting our hypothesis, these authors [64] demonstrated enhanced platelet aggregation responses to 5HT in 19 anaemic iron deficient patients (Hb 8.79±0.24 g/dl; serum iron 3.8±0.32 µmol/l) compared to controls. [64] The aggregation response corrected in all patients retested following treatment of their iron deficiency. [64] 5HT was examined because iron deficiency impaired the activity of the iron-containing platelet monoamine oxidase that metabolises 5HT; resuspension studies confirmed altered aggregation responses were due to the platelet, not the plasma.[64]

**Figure 3:**
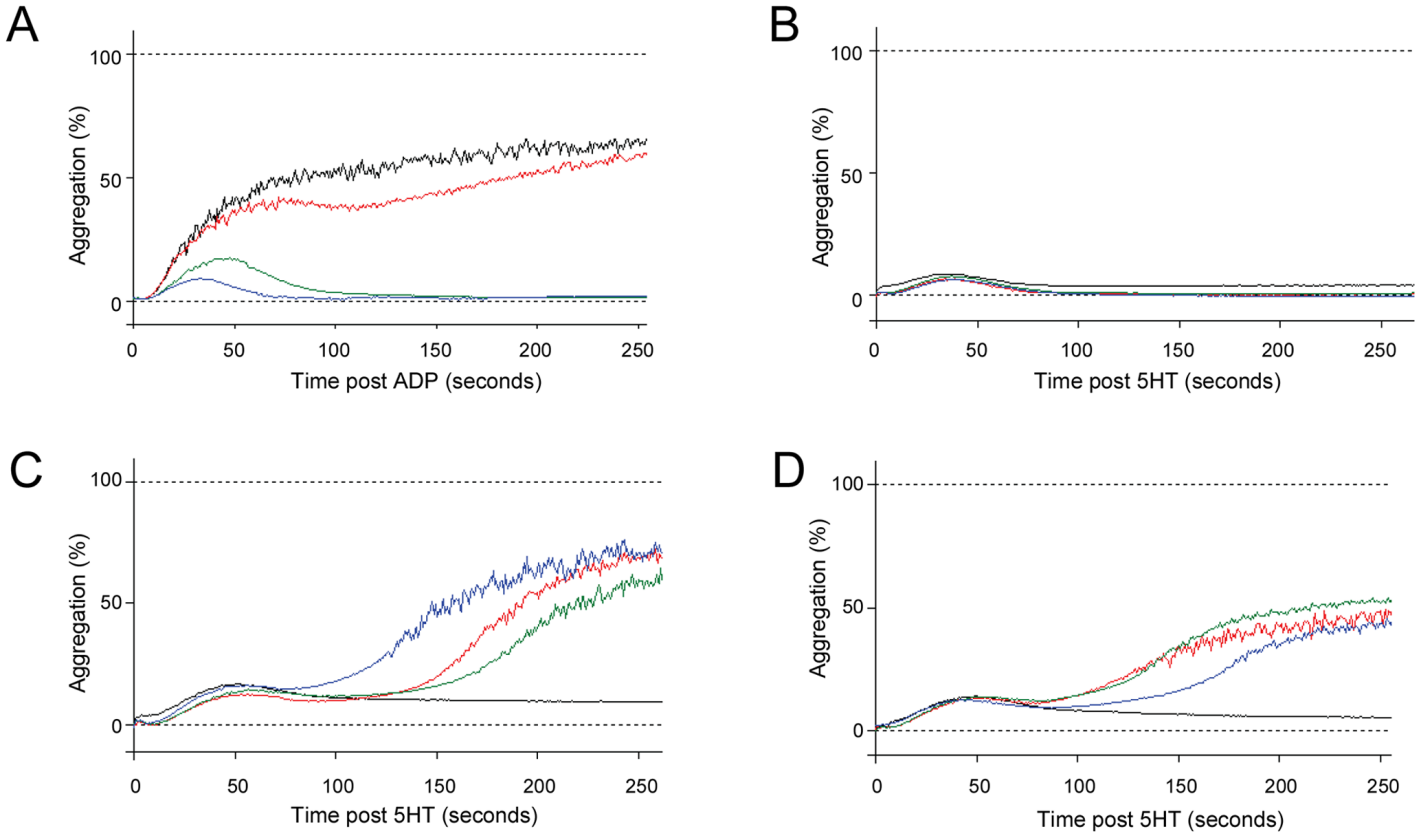
Representative platelet dose response curves. **A**) Typical control and iron deficient responses to ADP at 5 (blue), 10 (green), 20 (red) and 50 (black)µM. **B**) Typical control dose response curves to 5HT at 20 (blue), 200 (green), 2,000 (red) and 20,000 (black) µM–note the absence of the secondary wave of aggregation. **C**) Representative 5HT dose response curves displaying delayed secondary wave of aggregation observed in all severely iron deficient patients (hemoglobins 5.0–7.5 g/dl). The traces illustrated (5HT at 20 (blue), 200 (green), 2,000 (red) and 20,000 (black)) µM were from an individual with ferritin 4 µg/L, iron 3 µmol/l, hemoglobin 7.5 g/dl. **D**) Traces from the same individual as in (**C**), following a 6 month course of iron that resulted in improved iron indices (ferritin 31 µg/L, iron 7 µmol/l, hemoglobin 10.5 g/dl). Note that despite further treatments, iron deficiency persisted due to ongoing hemorrhagic losses. Aggregation curves are displayed for 5HT at 20 (black), 200 (green), 2,000 (red) and 20,000 (blue)µM.

These findings were replicated in a carefully selected subgroup of patients. Controls displayed secondary aggregation waves to ADP (Figure 3A), but not 5HT (Figure 3B). In contrast, severely iron deficient patients displayed delayed secondary aggregation waves to 5HT, (Figure 3C) partially correcting with improved iron indices (Figure 3D).

**Figure 4:**
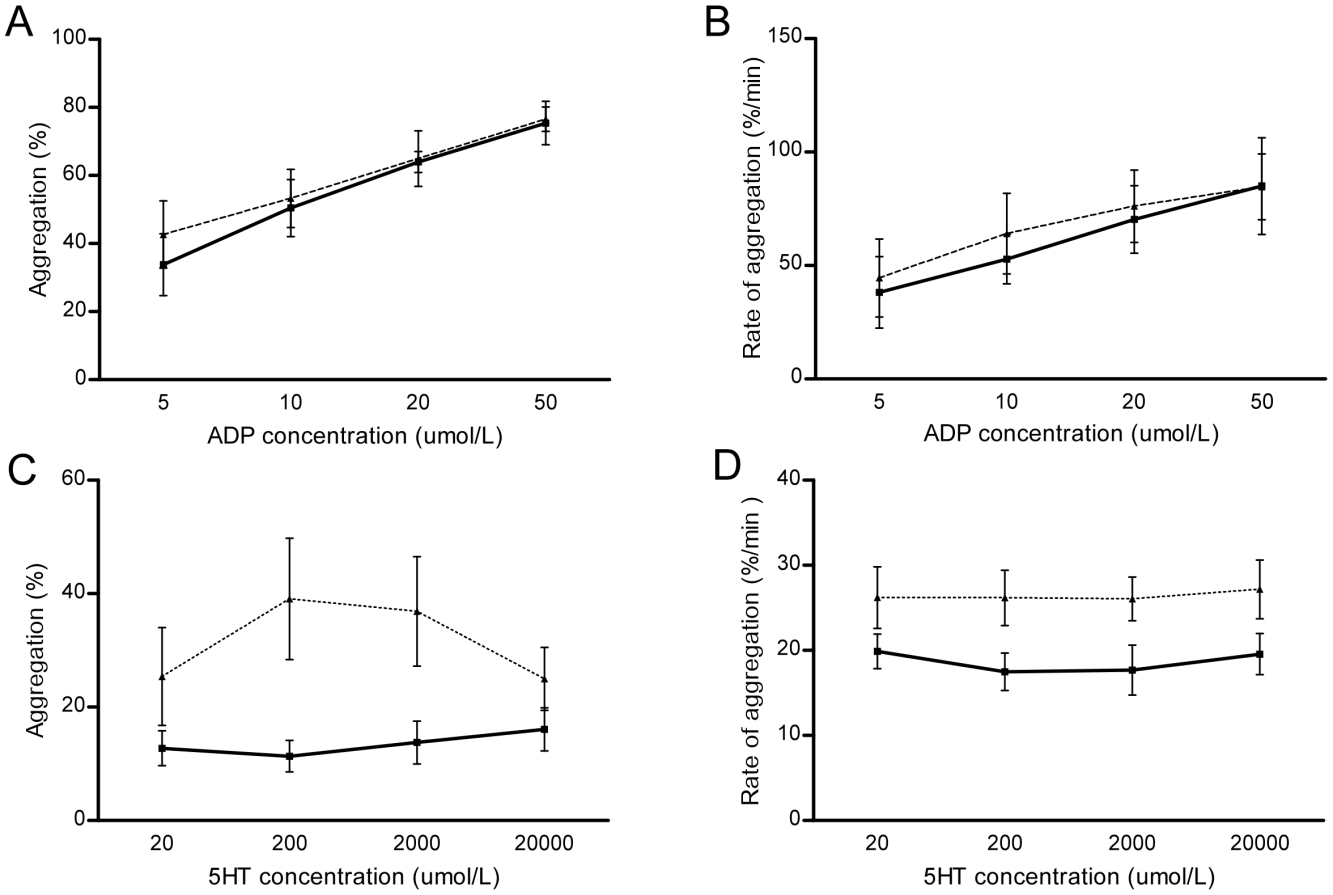
Comparison of platelet dose response curves in response to agonists in iron deficient patients and controls. Solid lines indicate controls; dotted lines represent the iron deficient group. Error bars represent standard error of the mean. **A**) Total aggregation in response to ADP at 5–50 µmol/L. **B**) Rate of aggregation in response to ADP. Since circulating blood should spend less than two seconds between pulmonary transits, the rate of aggregation may be particularly relevant. **C**) Total aggregation in response to 5HT. **D**) Rate of aggregation in response to 5HT.

To examine further, participants were assigned to two groupings defined by serum ferritin: iron deficient (ferritin 2–17 (median 8)µg/L, n = 7) and controls (ferritin 24–98 (median 38)µg/L, n = 8). No individual had a ferritin between 17 and 24 µg/L. ADP induced similar dose-dependent aggregation in iron deficient patients and controls (Figure 4A, Figure 4B). However, iron deficient HHT patients displayed enhanced total aggregation to 5HT over a five minute period (Figure 4C). The iron deficient group also displayed faster rates of aggregation in response to 5HT (Figure 4D).

The variables of 5HT aggregation and aggregation rate were skewed and normalised by log(ln) transformation (SFigure2). In univariate and multiple regression, serum iron was inversely related to (ln)aggregation (Table 6A). Serum iron was also inversely related to (ln)rate of aggregation. These inverse associations persisted after adjustment for 5HT dose and ferritin (Table 6B). Using MANOVA to adjust for participant and 5HT concentration, either iron or ferritin explained 14% of the total variance in (ln)5HT aggregation rate (p = 0·039 (iron); p = 0.021 (ferritin)).

**Table 6:** Multiple regression analyses of 5HT-induced aggregation parameters.

	Regression coefficient	95% confidence intervals	P value
**A) (Ln) total aggregation**			
5HT (µmol/L)	0.000036	6.8×10^−6^, 0.000065	0.018
Ferritin (µg/L)	−0.027	−0.043, −0.011	0.002
Serum iron (µmol/L)	−0.082	−0.14, −0.028	0.005
Iron*ferritin (µg*µmol/L^2^):	0.0012	8.7×10^−6^, 0.0024	0.052
**B) (Ln) rate of aggregation**			
Ferritin (µg/L)	−0.0062	−0.010, −0.0022	0.004
Serum iron (µmol/L)	−0.040	−0.061, −0.019	0.001

**A**) The distribution for aggregation achieved was skewed and normalised by log transformation (Figure S2A). (Ln)aggregation was therefore used as the dependent variable for regression. A model restricted to first order variables was not as strong as the final model including the iron-ferritin interaction term (iron*ferritin (µg*µmol/L^2^)). This model of 24 assays explained 72% of the variance of (ln)aggregation (p = 0.0001). **B**) The distribution for rate of aggregation was skewed and normalised by log transformation (Figure S2B). (Ln)rate of aggregation was therefore used as the dependent variable for regression. Final model for (ln)rate of aggregation in all 22 available assays, in a model that explained 77.4% of the variance (p<0.0001).The crude coefficient with iron was similar at −0.036 [95% CI −0.053, −0.0187], p<0.0001. There was no relationship with 5HT concentration in univariate or iron/ferritin- adjusted regression (data not shown).

## Discussion

In this study, paradoxical embolic strokes associated with the right-to-left shunts of CT-evident PAVMs were shown to be associated with low serum iron levels which approximately doubled age-adjusted stroke risks. Although there are multiple metabolic and physiological associations with iron deficiency, and the current study does not demonstrate causality, platelet data presented here and four decades ago, [64] highlight one consequence of iron deficiency that would be highly relevant to stroke pathogenesis, namely enhanced platelet aggregation responses.[71]

Strengths of the current study include the lack of confounding inflammatory diseases, and consistent assessment methodologies including standardised timings of blood sampling which is important because iron levels vary during the day. The absence of arterial blood gas measurements of PaO_2_ may be considered a weakness, but the reproducibility of the replicate pulse oximetry SaO_2_ measurements (Table 1) render this criticism less important. The similar haematinics defined by low serum iron, and the ferritin values conventionally used to define iron deficiency (Table 2), support the use of both low serum iron and low ferritin as markers of iron deficiency in the cohort in which ferritin levels are often difficult to interpret due to concurrent conditions, [61] [62] particularly hepatic AVMs in HHT patients [60].

The data extend the previous studies on stroke risk in PAVM patients, effectively analysing patients already within the higher shunt grade shown by Velthius and colleagues to be associated with enhanced risk of paradoxical embolic events. [18] Echocardiographic measurements were not made on the study population, but the majority of patients in this study would be expected to have a Grade 3 shunt, because they had PAVMs which were visible on CT scans. Iron deficiency has not been proposed previously as a risk factor for paradoxical embolic strokes in PAVM patients. However, in the general population, the CVDSACTS cohort study of 1,772 adults over 40 years, [72] NHANES I subgroup of 1,039 white women aged 45–74 ys, [73] and KLoSHA study of 965 Koreans aged over 65 years [74] all support an epidemiological link between iron deficiency and ischaemic stroke risk operating independently to known ischaemic stroke risk factors, even if this was not the primary focus of the respective reports.[72] [74]

Iron deficiency in HHT results from under-replacement of iron losses rather than the severity of haemorrhage *per se*, [34] but will be more common in patients with greater haemorrhagic burdens and therefore vascular damage. There are also innumerable consequences of iron deficiency including anaemia/reduced blood oxygen content, [51] high cardiac output with lower systemic vascular resistance, [75] [76] [77] and increased blood viscosity. [78] Additionally, in the HHT population, we recently demonstrated that iron deficiency was associated with VTE, and elevated Factor VIII. [60] We cannot exclude a role for these or other processes in the pathogenesis of paradoxical embolic stroke, though note there was no increase in VTE in patients with ischaemic stroke, and no clear associations between ischaemic stroke and polycythaemia, Factor VIII, or coagulation parameters that would be shortened in venous prothrombotic states (Table 3, Table 5). In the current study, there was an unexpected association between ischaemic stroke and higher concentrations of fibrinogen, the predominant circulating plasma protein for platelet adhesion. [69] In general population studies, depletion of fibrinogen is “promising” in the treatment of acute ischaemic stroke. [79] In the current study, the strokes resulted in only partial anterior or posterior circulation syndromes. Taken together with the known efficacy of anti-platelet agents in stroke prevention, [70] the findings point towards a process influenced by paradoxical emboli of small platelet aggregates in circulating blood.

There are few relevant data on circulating platelet aggregates, [80] but platelet studies in the general population have demonstrated higher spontaneous platelet aggregation in ischaemic stroke patients. [81] The original association between iron deficiency and enhanced aggregation to 5HT [64] has however, escaped notice: PubMed searches in November 2013 for “iron,” “platelet(s),” and {“5HT” or “serotonin”} retrieved no relevant results. 5HT is usually studied with respect to neurotransmission, following release from nerve tissue but is also released from the dense granules of platelets and intestinal enterochromaffin cells. [69] [71] 5HT plasma concentrations increase after platelet activation, intestinal ischaemia/reperfusion, drug administration, and in a number of disease states including atherosclerosis. [69] [71] [80] [82] [83] The importance of 5HT-platelet interactions remains controversial as in the West, 5HT is considered a minor platelet agonist. However, evidence particularly from Japan suggests greater importance: Antagonists to the receptor 5HT2A result in dose-dependent inhibition of platelet aggregation in ischaemic stroke patients, [80] and achieved therapeutic equivalence with aspirin in secondary prevention of ischaemic stroke. [83] Conversely, cardiovascular side effects are recognised for drugs that increase extracellular 5HT/serotonin.[84]

The presented and published [64] data support a model in which iron deficiency enhances platelet aggregation. We cannot confirm a causal role to stroke pathogenesis- for example, it is conceivable that iron deficiency is a marker of more severe vascular damage that could be promoting platelet aggregation *in vivo*, in addition to the vessel-independent effects observed with iron deficient platelets *ex vivo*. Nevertheless, irrespective of the precise mechanisms of formation, a proportion of platelet aggregates which should lodge safely in pulmonary capillaries/arterioles after forming or entering the venous circulation, would escape pulmonary capillary filtration via the right-to-left shunts provided by PAVMs, and instead occlude systemic arterioles/capillaries including those of eloquent regions of the brain (Figure 1C–F). The final outcome following impaction will depend on local responses including orchestration of thrombo-inflammatory cascades, [69] but during any thrombus resolution, systemic tissues do not benefit from a separate arterial supply of oxygenated blood, as in the lung.[1] [3]

Our data do not allow us to say whether the stroke risk findings would be more important than grading with contrast echocardiography - further studies could be conducted to assess if the iron deficiency risk is greater in echo- stratified patients. However, for patients with CT-proven PAVMs, the key biomarker for ischaemic stroke risk appears to be iron deficiency, and not the severity of right-to-left shunt. Iron deficiency is potentially reversible if iron intake can be increased to meet demands,[34] and reassuringly, in the current study, use of iron tablets to treat iron deficiency due to HHT-related bleeding [34] was not associated with enhanced stroke risk (Table 3, Table 5). Correction of iron deficiency is however often difficult in HHT patients, due to ongoing haemorrhagic blood losses. [22] [23] [24] [34] The additional data, albeit weak, that higher SaO_2_ is associated with a lower stroke risk further supports the need for therapeutic embolisation to reduce shunt size, while providing some reassurance to individuals with smaller, untreatable, but CT-detectable PAVMs.

In summary, the data indicate that iron deficiency and circulating platelets provide potentially novel opportunities for targeted stroke reduction strategies that could be examined in future clinical trials, particularly in the setting of compromised pulmonary capillary filtration.

## Supporting Information

Figure S1 **Quadratic regression plots for continuous variables associated with stroke risk.** Quadratic regression plots (with shaded intervals representing the 95% confidence intervals) for continuous patient variables versus ischaemic stroke risk for **A**) Serum iron; **B**) SaO_2_. Note the near-linear regression lines, particularly for serum iron.
(TIF)

Figure S2 **Normal quantile plots for platelet aggregation parameters used as dependent variables in regression analyses.** A) Total aggregation across all concentrations of 5HT. Note skewed distribution. **B**) Rate of aggregation across all concentrations of 5HT. Note skewed distribution. **C**) Log-transformed total aggregation. **D**) Log-transformed rate of aggregation.
(TIF)
