# Supplementary figures and images for: Ischaemic Strokes in Patients with Pulmonary Arteriovenous Malformations and Hereditary Hemorrhagic Telangiectasia: Associations with Iron Deficiency and Platelets
Source: PLoS One. 2014 Feb 19;9(2):e88812. doi: 10.1371/journal.pone.0088812 (PMC3929507; doi:10.1371/journal.pone.0088812)

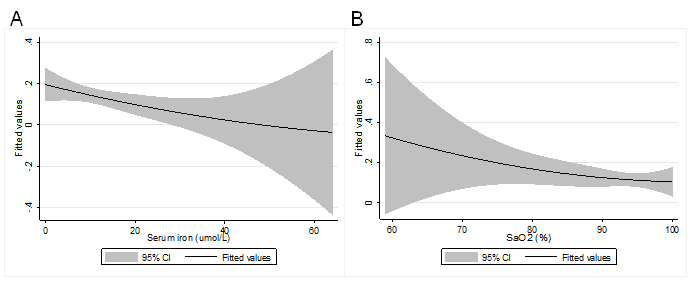

Supplement: Figure S1 — Quadratic regression plots for continuous variables associated with stroke risk. Quadratic regression plots (with shaded intervals representing the 95% confidence intervals) for continuous patient variables versus ischaemic stroke risk for A) Serum iron; B) SaO2. Note the near-linear regression lines, particularly for serum iron. (TIF) [file pone.0088812.s001.tif]

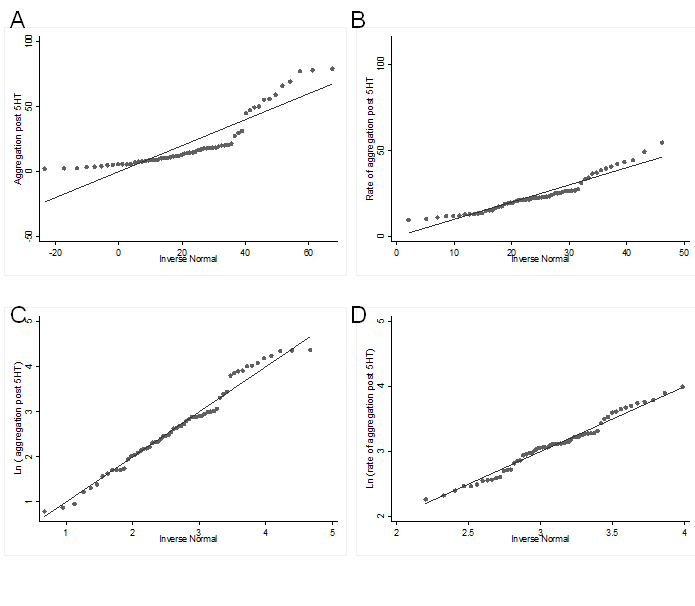

Supplement: Figure S2 — Normal quantile plots for platelet aggregation parameters used as dependent variables in regression analyses. A) Total aggregation across all concentrations of 5HT. Note skewed distribution. B) Rate of aggregation across all concentrations of 5HT. Note skewed distribution. C) Log-transformed total aggregation. D) Log-transformed rate of aggregation. (TIF) [file pone.0088812.s002.tif]
